# Arachnoid Cyst in Middle Cranial Fossa With Intraorbital Cyst (Orbital Meningocele)

**DOI:** 10.7759/cureus.18795

**Published:** 2021-10-15

**Authors:** Vilas M Kulkarni, Sachin B Chitalkar, Sanjay M Khaladkar, Rahul S Navani, Purnachandra Lamghare

**Affiliations:** 1 Radiodiagnosis, Dr. D. Y. Patil Medical College, Hospital and Research Center, Pune, IND

**Keywords:** orbital meningocele, orbital cyst, intra-orbital cyst, intra-orbital meningocele, arachnoid cyst

## Abstract

Intracranial arachnoid cysts are extra-axial non-enhancing cerebrospinal fluid (CSF) density lesions. These are usually incidental findings on radiological investigations. Usually, the patients with arachnoid cysts are asymptomatic until the cyst grows large while symptomatic patients present with headaches, seizures, and focal neurological deficits. The adjacent calvarial bone may show remodeling and scalloping. Magnetic resonance imaging (MRI) stands superior in soft-tissue contrast and multiplanar imaging in excluding other lesions from the arachnoid cyst. Arachnoid cysts follow CSF signals in all pulse sequences with no gadolinium enhancement. Intraorbital extension of the intracranial arachnoid cyst (intraorbital meningocele) is rarely reported in the literature and occurs through the small bony defect. We report a case of a 20-year-old male presenting with proptosis who was detected to have an arachnoid cyst in the middle cranial fossa with intraorbital extension through a small bony defect in the lateral wall of orbit with the resultant orbital cyst.

## Introduction

Arachnoid cysts (ACs) are relatively common benign and mostly asymptomatic developmental anomalies occurring in association with the central nervous system, within the intracranial compartment, or the spinal canal. They are thought to arise due to the congenital separation of the arachnoid layer with an accumulation of cerebrospinal fluid (CSF) within the formed potential space. Most of the ACs are detected incidentally when investigated for other complaints. These cause symptoms due to the large size and mass effect on adjoining structures [1]. Several cystic and cyst-like isolated orbital lesions may be encountered in imaging of the orbits including developmental cysts such as dermoid cysts, epidermoid, teratoma, congenital cystic eye; and acquired cysts such as abscess, hematoma, lacrimal gland cyst, lymphangioma, and hydatid cyst [2]. We present a case of the intracranial AC with extension through a defect in the lateral wall of the left orbit causing the intraconal retrobulbar AC.

## Case presentation

**Figure 1:**
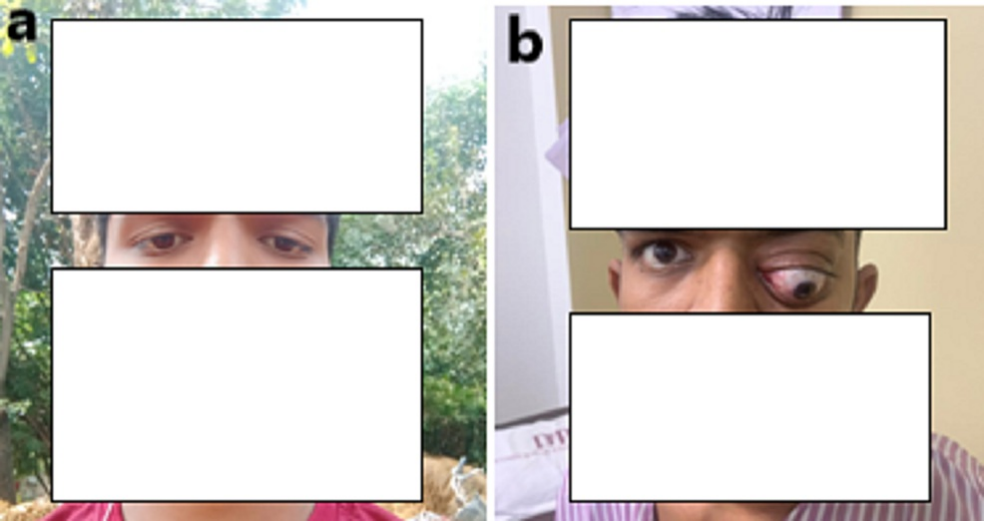
(a) Frontal view showing normal left eye six months back. (b) Frontal view during present hospital admission with proptosis of the left eye.

A 20-year-old male, a previously asymptomatic patient with normal vision, presented to our outpatient department with diplopia, decreased visual acuity, and gradually increasing painless protrusion of the left eye for the last four months. Conjunctival congestion was present due to incomplete closure of eyelids (Figures 1a, 1b).

It was associated with left-sided hemicranial headache. It was not associated with vomiting, altered sensorium, seizures, slurred speech, and limb weakness. There was no history of weight loss, appetite loss, cough, seizures, limb weakness, any skin changes, surgery, drug intake, or close contact with pets. There were no co-morbid conditions like diabetes mellitus, hypertension, or tuberculosis. There was no history of orbital trauma. On examination, visual acuity in the left eye was 6/20. There was proptosis in the left eye with a downward and outward deviation of the eye. There was no change of proptosis on straining (Valsalva maneuver). No lid lag, retraction, or edema was present. Restricted upward movement of left eye-ball was noted. On palpation pulsation or thrill was absent. Corneal sensitivity was normal with cotton wisp. There was no regional lymphadenopathy. Systemic examination was unremarkable.

**Figure 2:**
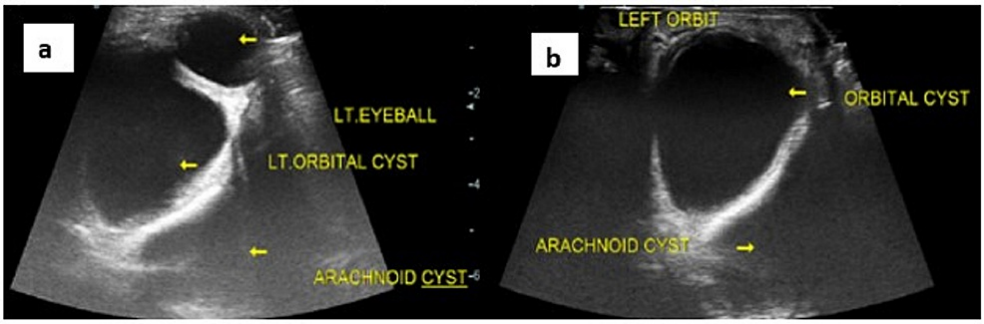
(a) Ultrasound examination of the left orbit showing well-defined anechoic cystic lesion without solid component, septations, or calcification and intracranial cyst behind the left sphenoid wing without solid component, septations, or calcification. (b) Ultrasound examination cranially to the previous image showing intraorbital cyst and intracranial cyst in the left middle cranial fossa.

On orbital ultrasound examination, a well-defined thin-walled anechoic cystic lesion was noted in the left intraorbital region causing proptosis of the left eye. No internal echoes, septations, calcification, or any solid component was noted. Another well-defined thin-walled anechoic cystic lesion was noted in the left middle cranial fossa behind the left orbit. On ultrasound examination, no obvious demonstrable communication was noted between the two cysts (Figures 2a, 2b). 

**Figure 3:**
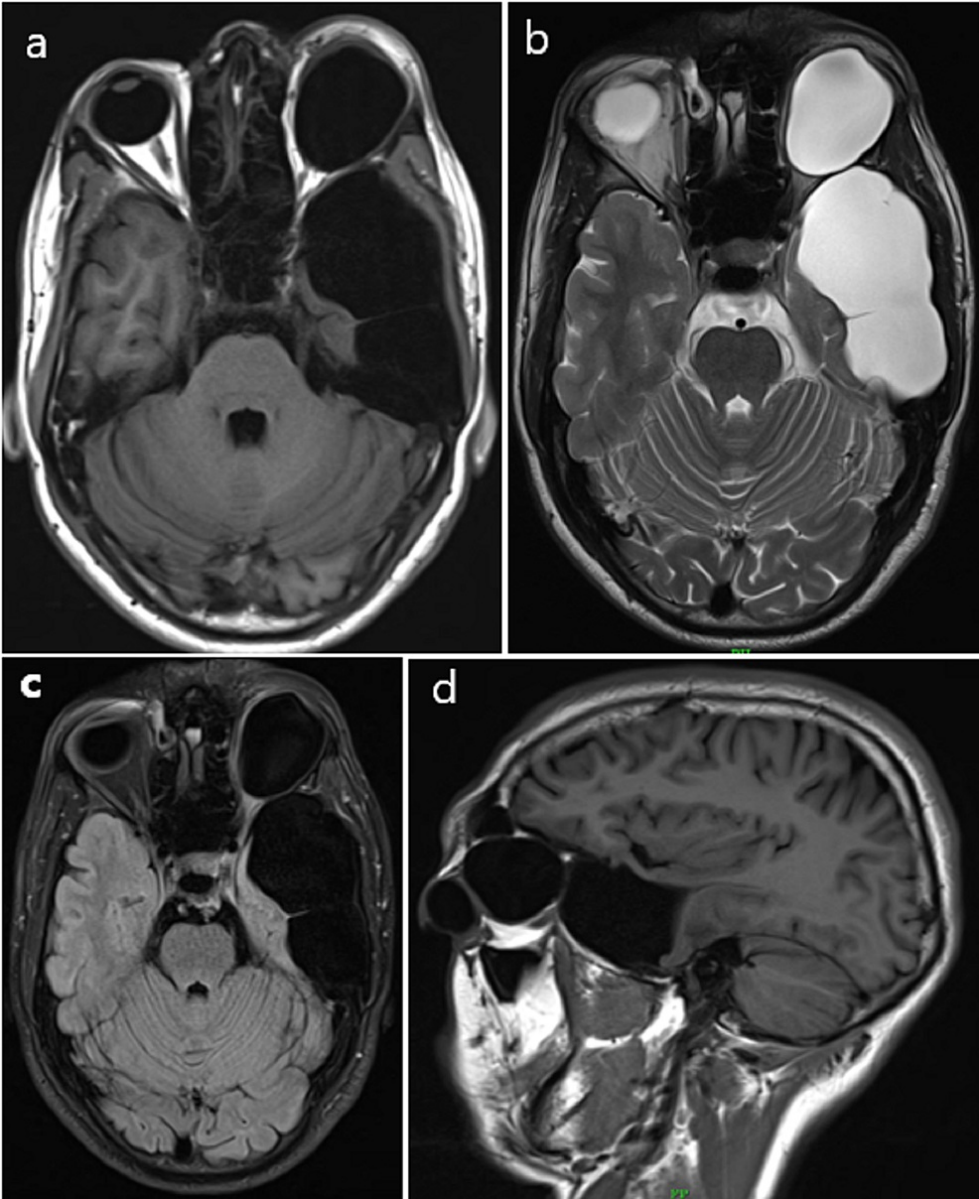
(a) Axial T1WI. (b) Axial T2WI. (c) FLAIR axial. (d) Sagittal T1 images showing CSF signal intensity extra-axial cystic lesion in the left middle cranial fossa and intraorbital cystic lesion.

**Figure 4:**
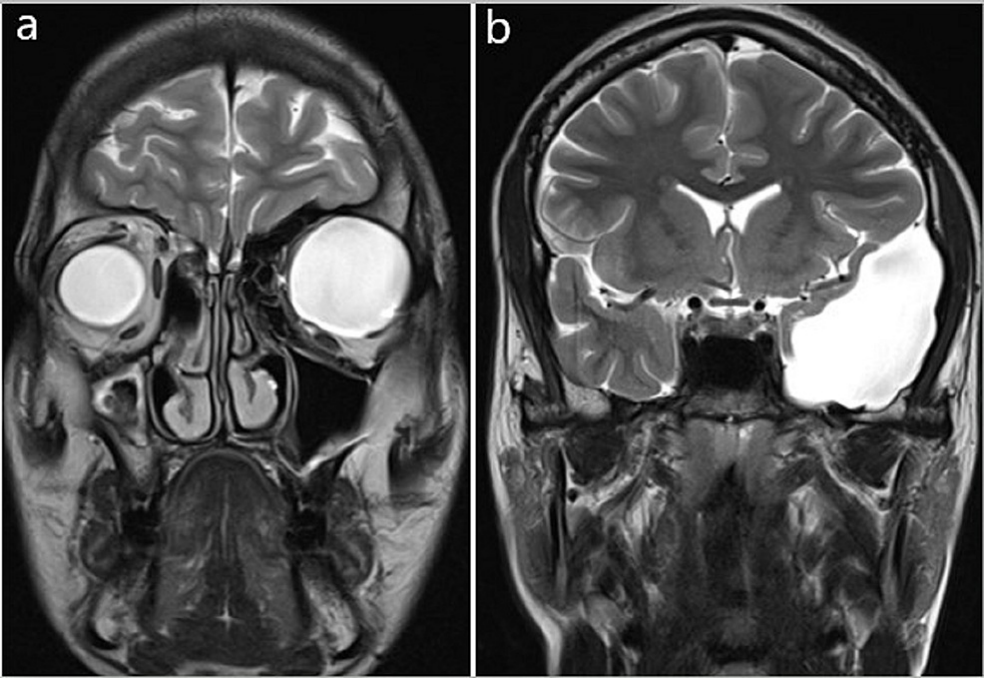
(a) Coronal T2WI through orbit. (b) Coronal T2WI through middle cranial fossa showing intraorbital cystic lesion and extra-axial cystic lesion in the left middle cranial fossa, respectively.

**Figure 5:**
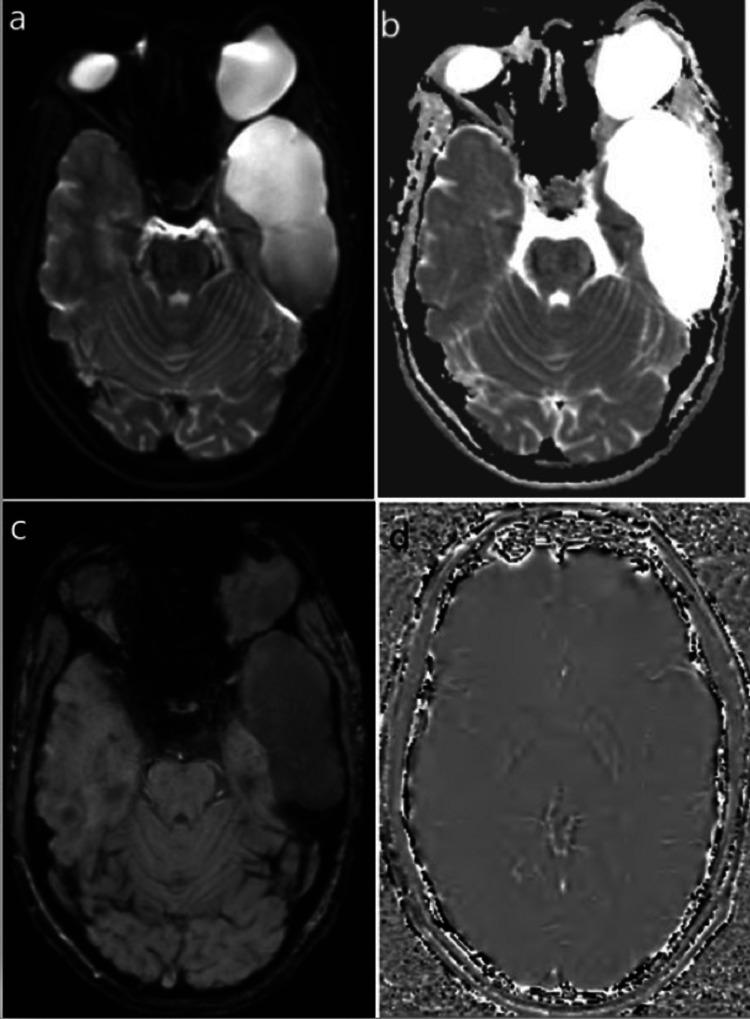
(a) Diffusion-weighted imaging (DWI) and (b) apparent diffusion coefficient (ADC) map showing no diffusion restriction in the intracranial or intraorbital lesion. (c) The magnitude and (d) phase images of SWI showing no “blooming” artifact in the intracranial or intraorbital lesion.

Magnetic resonance imaging (MRI) of the brain and orbits showed thin-walled large well-defined CSF signal intensity extra-axial cystic lesion measuring approximately 76x43x49 mm (anterior-posterior x transverse x cranio-caudal, respectively) in left middle cranial fossa causing scalloping of the left sphenoid wing and inner table of left squamous temporal bone and floor of the left middle cranial fossa. It was isointense to CSF on T1 weighted image (T1WI), T2 weighted image (T2WI), and fluid-attenuated inversion recovery (FLAIR) image without diffusion restriction on diffusion-weighted imaging (DWI). No hemorrhage was noted on the Susceptibility weighted imaging (SWI) (Figures 3a-3d, 4a, 4b, 5a-5d).

**Figure 6:**
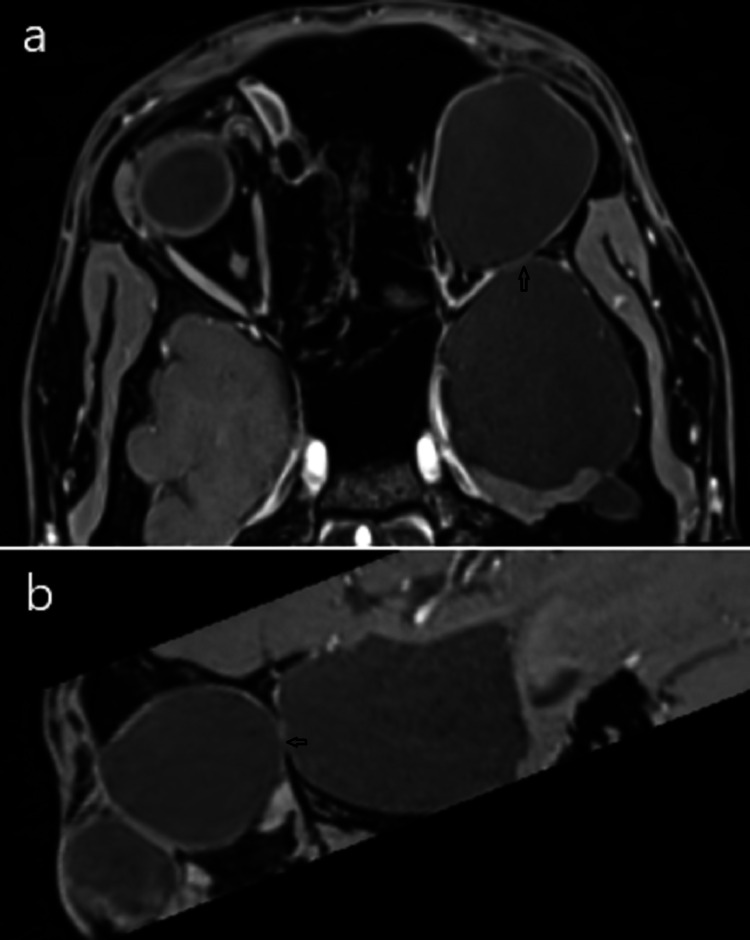
(a) T1 VIBE axial and (b) T1 VIBE sagittal oblique images showing the bony defect (black arrow) along posterior-lateral bony orbital wall communicating intraorbital and intracranial cysts. VIBE - volumetric interpolated breath-hold examination

**Figure 7:**
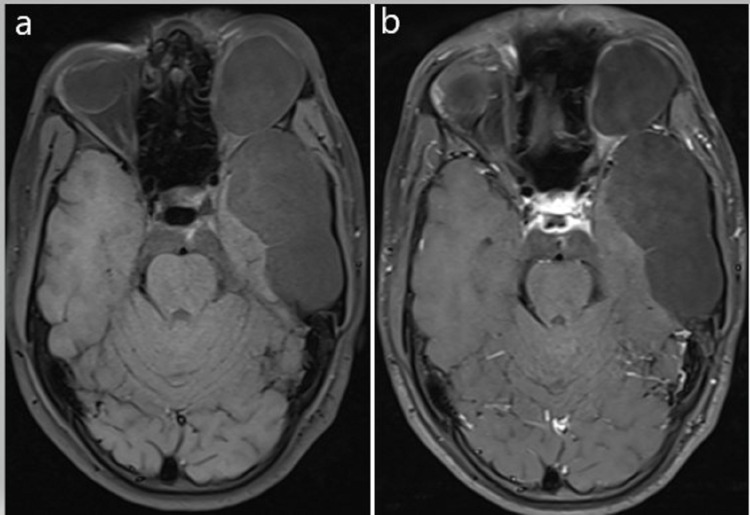
(a) T1 fat-saturation pre-contrast and (b) T1 fat-saturation post-contrast images showing no abnormal contrast enhancement.

It showed few thin septations and was causing mass effect on adjoining left temporal lobe parenchyma which was compressed and displaced medially and superiorly. No abnormal post-contrast enhancement was noted. A small bony defect of size 10x8 mm (transverse x cranio-caudal) was noted in the adjoining left sphenoid bone seen on T1 Volumetric interpolated breath-hold examination (VIBE) sequence (Figures 6a, 6b, 7a, 7b).

A well-defined CSF signal intensity cystic lesion measuring approximately 41x31x34 mm (anterior-posterior x transverse x cranio-caudal, respectively) was noted in left orbit inferior to left orbital roof causing extrinsic compression and inferior displacement of superior rectus and optic nerve. Mass effect was noted on the left eyeball which was compressed and displaced anteriorly and inferiorly with resultant proptosis (Figures 3a, 3b, 6a, 6b). No post-contrast enhancement was noted (Figures 7a, 7b). The diagnosis of AC in the left middle cranial fossa and left orbital meningocele with communication between the two cysts through a small bony defect in the left lateral wall of orbit was given.

**Figure 8:**
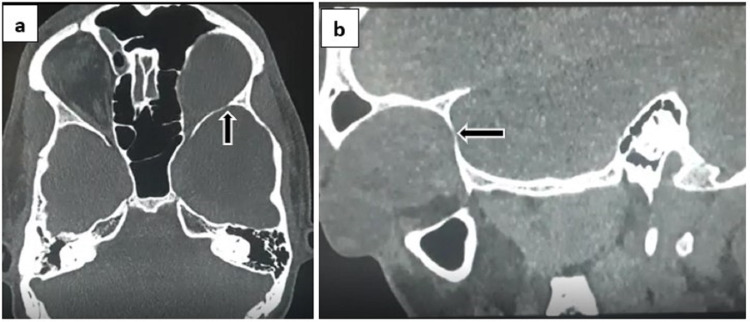
(a) CT axial section and (b) reformatted sagittal oblique view showing scalloping of left sphenoid wing with a small bony defect (black arrow) communicating intraorbital and intracranial cystic lesions.

Non-contrast enhanced computed tomography (CT) scan of orbits was performed to evaluate the bony changes. CT revealed scalloping of the left orbital roof and lamina papyracea, scalloping of the left sphenoid wing and floor of left cranial fossa, and a small defect in the left lateral wall of the orbit (Figures 8a, 8b).

## Discussion

Intracranial ACs are extra-axial non-enhancing CSF density lesions on CT scans. The adjacent calvarial bone may show remodeling and result in hypoplasia to the adjoining brain parenchyma, usually in the middle cranial fossa [1]. ACs frequently have communication with the subarachnoid space making up 1% of the intracranial space-occupying lesions (SOL), and although typically seen in children may remain undiagnosed until adulthood. These can be supratentorial (90%) or infratentorial (10%). Among supra-tentorial ACs, temporal (60%) AC forms the majority of cases. Most of these can be asymptomatic, however, can present with headaches if large. Other presentations can be vomiting, hydrocephalus, subdural hemorrhage, failure of upward gaze, focal neurological deficits, and seizures [3,4].

Diagnostic evaluation includes identification of intracranial ACs, detection of mass effect, and complications if any. Investigations for ACs are neurosonography, plain or contrast CT, MRI [5]. MRI has advantages of superior soft-tissue contrast and multiplanar imaging in excluding other lesions from the AC. ACs follow CSF signals in all pulse sequences with no gadolinium enhancement.

Differential diagnoses of intraorbital cystic mass are lesions arising from orbit, globe, lacrimal system, paranasal sinuses, or meninges. Developmental lesions can be dermoid and epidermoid cysts or coloboma. Acquired pathologies can be orbital abscesses, mucocele, and vascular malformations like arteriovenous malformation [6]. AC of the optic nerve has been reported in few case reports (three case reports on the literature review on Pubmed). The radiologist plays an important role in the management of intraorbital lesions with quick diagnosis based on characteristic imaging patterns on ultrasound, CT, or MRI.

Intraorbital extension of intracranial AC (intraorbital meningocele) has been very rarely reported in the literature [7]. However, the concomitant presence of intracranial - temporal lobe cystic lesion and the intraorbital cystic lesion should raise suspicion of the intracranial AC with intraorbital meningocele. Careful identification of the bony defect may be done using different radiological modalities. If the defect is large enough, orbital ultrasonography (USG) may be helpful to demonstrate the continuity of the intracranial cyst to the orbital cyst [8]. MRI is helpful in the characterization of the intracranial and intraorbital lesions. It has limited value in the demonstration of the bony defect. A plain CT scan (thin sections at bony algorithm) is the gold standard in the demonstration of the bony defect.

Intraorbital meningocele can be congenital or post-traumatic constituting 1%-1.5 % of all meningoceles. Congenital meningoceles are usually treated by surgical excision or ligation of the cyst along with the closure of the defect. If the defect is large, it can be closed by titanium plates and microscrews or bone graft [9].

The vast majority of ACs are asymptomatic and conservative management is instituted for most of the patients. Surgical management is fraught with morbidity and avoided in asymptomatic patients not demonstrating signs of increased intracranial pressure or focal neurological signs. These asymptomatic patients need interval follow-up with CT and MRI to monitor growth and early recognition of complications [10,11].

Symptomatic cysts manifesting with seizures, hydrocephalus, increased intracranial pressure, neurological deficits, and those complicated by intracystic or subdural hemorrhage are usually managed surgically. Few authors believe that all ACs exert a mass effect and should be managed surgically regardless of symptomatology to avoid potential complications such as compression on adjoining brain structures, cyst rupture, and intracystic or subdural bleed. The relationship of the cyst to adjoining brain structure should be carefully explored before endeavoring surgical exploration [11,12].

Intraorbital extension of the intracranial AC (intraorbital meningocele) is one such indication for operative management. Our patient presented with severe proptosis with visual impairment, hence an ideal candidate for surgical intervention. Surgical management of the ACs includes procedures such as surgical excision, cystoperitoneal shunting, endoscopic ventriculo-cystostomy or ventriculo-cystocistenostomy, stereotactic cyst-ventricular shunting, and stereotactic intracavitary irradiation [10-13]. Our patient underwent left temporo-zygomatico-orbital craniectomy with intracranial and intraorbital cyst excision. Post-operatively, the patient developed pneumocephalus and thin subdural hematoma in the left temporal region and pneumocephalus in the left frontal region, which regressed on subsequent scans. Left eye proptosis resolved with normal vision in the post-operative period.

## Conclusions

ACs are relatively common benign intracranial anomalies while associated intraorbital cystic lesions are not a common occurrence. The concurrent presence of both AC and the intraorbital cystic lesion should raise the suspicion of intraorbital extension of the AC and an active search for the bony defect along the orbital wall should be made to confirm the diagnosis of orbital meningocele. The bony defect can be detected on CT and if large on USG and MRI. Our case highlights the utility of both MRI and CT in the detection of a small bony defect in the lateral wall of orbit with resultant intraorbital meningocele in the case of AC in the left middle cranial fossa.
